# Supplementary material for: The prevalence and associated factors of adverse pregnancy outcomes among Afghan women in Iran; Findings from community-based survey
Source: PLoS One. 2021 Jan 15;16(1):e0245007. doi: 10.1371/journal.pone.0245007 (PMC7810300; doi:10.1371/journal.pone.0245007)
Supplement: S1 File — (DOCX) [file pone.0245007.s001.docx]

| **Q.No.** | **Questions** | **Category Coding** | **Skip** |
| --- | --- | --- | --- |
| **1.** | How old are you? | _______ years |  |
|  |  |  |  |
| **2.** | How long have you been | _______ years |  |
|  | living in Iran? |  |  |
| **3.** | What is your resident status | Holding a valid visa……….1 |  |
|  | In Iran? | Others ……………………..2 |  |
|  |  | *Please specify…..* |  |
| **4.** | Type of family you live | Nuclear ……………………1 |  |
|  | In | Joint………………………..2 |  |
|  |  | No. of Family members …… |  |
|  |  |  |  |
| **5.** | What is the highest level | Illiterate.……………………1 |  |
|  | of school you attended? | Primary…….. ….…………..2 |  |
|  |  | Secondary ...…..……………3 |  |
|  |  | High school ..……………....4 |  |
|  |  | College or higher……..…….5 |  |
| **6.** | How old were you when | _______years |  |
|  | you got married? |  |  |
| **7.** | How old is your | _______ years |  |
|  | husband ? |  |  |
| **8.** | Is your husband living | Yes ………………..…..….1 |  |
|  | with you? | No ………………….……..2 |  |
| **9.** | What is your husband | Afghan……………....…….1 |  |
|  | nationality | Iranian ……….…………....2 |  |
|  |  | Others ……………..………3 |  |
|  |  |  |  |
| **10.** | What is the highest level | Illiterate.……………………1 |  |
|  | of school your husband | Primary…….. ….…………..2 |  |
|  | attended? | Secondary ...…..……………3 |  |
|  |  | High school ..……………....4 |  |
|  |  | College or higher……..…….5 |  |
| **11.** | How many children do | _______ |  |
|  | you have? |  |  |
| **12.** | What is your present | Employed …………..…….1 |  |
|  | occupation? | Unemployed ...……………2 |  |
|  |  | Self-employed…………….3 |  |
|  |  | Others ……………………..4 |  |
|  |  |  |  |
| 13. | What is your husband | Employed ……………….1 |  |
|  | present occupation? | Unemployed ...……………2 |  |
|  |  | Self-employed…………….3 |  |
|  |  | Others ……………………..4 |  |
|  |  |  |  |
| 14. | How much is your | Rs.___________ |  |
|  | monthly family income? |  |  |
| 15. | Which is the nearest | Govt Sub center / Primary |  |
|  | healthcare facility from | Health Center …………........1 |  |
|  | your house? | Govt Hospital……………....2 |  |
|  |  | Private clinic / hospital …….3 |  |
|  |  | NGO………………………...4 |  |
| 16. | How far is the nearest | ________ kilometres |  |
|  | healthcare facility from |  |  |
|  | your house? |  |  |
| 17. | How do you travel to | Walk ………………………1 |  |
|  | this facility? | Public transport……………2 |  |
|  |  | Private vehicle..………..…..3 |  |
|  |  | Taxi ………………………..4 |  |
| 18. | Do you own any kind of | Yes…………………………1 |  |
|  | health insurance? | NO …………………………2 |  |
| 19. | How many times did you receive antenatal care during this pregnancy? | Number of times . . . . ….  Don't know . . . . . . . . . . . . . . . . . . . . . . 98 |  |

|  | **20.** | Have you ever experienced any of following conditions in your previous pregnancies? | History of preterm births:……………..1 |  |
| --- | --- | --- | --- | --- |
|  |  |  | Miscarriage or pregnancy loss at 20+  weeks.………………………………….2 |  |
|  |  |  | History of low birth weight (less than 2500 gram)……………………………..…….3 |  |
|  |  |  | History of gestational hypertension……4 |  |
|  |  |  | History of gestational diabetes……...…..5 |  |
|  |  | *(can mark* *multiple answers)* | History of any other conditions……...….6 |  |
|  |  |  | *Please specify………….;.* |  |
|  |  |  |  |  |

|  | **21.** | Have you experienced any of the following complications during recent pregnancy? | |  |  |  |
| --- | --- | --- | --- | --- | --- | --- |
|  |  |  |  |  |  |  |
|  |  |  |  |  |  |  |
|  |  |  |  | **Yes** | **No** | **Don’t know** |
|  |  |  |  | 1 | **0** | **98** |
|  |  | **21.1** | Preterm |  |  |  |
|  |  | Labor *(less than* | |  |  |  |
|  |  | *37weeks)* | |  |  |  |
|  |  | **21.2** | Abortion |  |  |  |
|  |  | /pregnancy loss at 20+ weeks | |  |  |  |
|  |  | **21.3** | Stillbirth |  |  |  |
|  |  |  |  |  |  |  |
|  |  | **21.4** | Eclampsia |  |  |  |
|  |  | / pre-eclampsia | |  |  |  |
|  |  | **21.5** | Early |  |  |  |
|  |  | rupture of membrane | |  |  |  |
|  |  | **21.6** | Gestational |  |  |  |
|  |  | hypertension (20+weeks) | |  |  |  |
|  |  | **21.7** | gestational |  |  |  |
|  |  | diabetes | |  |  |  |
|  |  | **21.8** | Intrapartum |  |  |  |
|  |  | Hemorrhage | |  |  |  |
|  |  | **21.9** | Infections |  |  |  |
|  |  |  |  |  |  |  |
|  |  | **21.10** | Other |  |  |  |
|  |  | complications | |  |  |  |
|  |  | *Please specify…..* | |  |  |  |
|  | **22.** | What was your child weight? | | Weight ………………. (gram)  I don’t know………………………….98 | |  |
|  |  |  |  |  |  |  |
|  |  |  |  |  |  |  |
|  | **23.** | At birth what was the size of the baby? | | Smaller than normal . . . . . . . . . . . . . . . . . . ... . 1  Normal . . . . . . . . . . . . . . . . . . . . . . . . . . . . .. . . 2  Larger than normal . . . . . . . . . . . . . . . . . … . . 3  Don't know . . . . . . . . . . . . . . . . . . . . . . . . . . . . .98 | |  |
|  |  |  |  |  |  |  |
|  | **24.** | Was your child healthy? | | Yes……………………………..1 | |  |
|  |  |  |  | No ……………………………...2 | |  |
|  |  |  | | In case of disease, please  specify………. | |  |
|  |  |  | |  |  |  |

|  | **Q.No** | **Questions** | **Category Coding** | **Skip** |
| --- | --- | --- | --- | --- |
|  | **25.** | Have you ever been  physically, sexually,  emotionally, or verbally  abused by your partner  or someone close to you during your pregnancy? |  |  |
|  |  |  | Yes…………………………….1 |  |
|  |  |  |  | If “No” skip to  Q.42 |
|  |  |  | No …………………………….2 |  |
|  |  |  |  |  |
|  |  | **25.1.** In the last year, have you been hit, slapped, kicked, or otherwise physically hurt by someone? | Yes…………………………….1 |  |
|  |  |  |  |  |
|  |  |  | No …………………………….2 |  |
|  |  |  |  |  |
|  |  | **25.2.** Have you ever been hit, slapped, kicked, or otherwise physically hurt by someone during pregnancy? | Yes…………………………….1 |  |
|  |  |  |  |  |
|  |  |  | No …………………………….2 |  |
|  |  |  |  |  |
|  |  | **25.3.** Within the last year, has anyone made you do something sexual that you did not want to do? | Yes…………………………….1 |  |
|  |  |  |  |  |
|  |  |  | No …………………………….2 |  |
|  |  | **25.4.** Are you afraid of your | Yes…………………………….1 |  |
|  |  | partner or anyone else? | No …………………………….2 |  |
|  |  | **25.5.** Does your partner ever humiliate you? Shame you? Put you down in public? Keep you from seeing friends or from doing things you want to do? |  |  |
|  |  |  | Yes…………………………….1 |  |
|  |  |  |  |  |
|  |  |  | No …………………………….2 |  |
|  |  |  |  |  |
|  |  |  |  |  |
|  | **26.** | Do you feel unsafe where you live? | Yes…………………………….1 |  |
|  |  |  | No …………………………….2 |  |
|  | **27.** | During the past month, did you miss any meals, not eat when you were hungry, because there was not enough food or money to buy food? |  |  |
|  |  |  | Yes…………………………….1 |  |
|  |  |  |  |  |
|  |  |  | No …………………………….2 |  |
|  | **28.** | Have you had any housing problems in last year? | Yes…………………………….1 |  |
|  |  |  | No …………………………….2 |  |
|  | **29.** | Have you had problems with depression or received counseling or medications for mental health concerns? | Yes…………………………….1 |  |
|  |  |  |  |  |
|  |  |  | No …………………………….2 |  |
|  |  |  |  |  |
|  | **30..** | During the past month, have you had little interest in doing things, or have you been bothered by feeling down, depressed, or hopeless? |  |  |
|  |  |  | Yes…………………………….1 |  |
|  |  |  |  |  |
|  |  |  | No …………………………….2 |  |
|  |  |  |  |  |
|  | **31.** | Is there anyone whom you count on when you need help? | Yes…………………………….1 |  |
|  |  |  | No …………………………….2 |  |
